# Role of PDGF-A/B Ligands in Cardiac Repair After Myocardial Infarction

**DOI:** 10.3389/fcell.2021.669188

**Published:** 2021-08-25

**Authors:** Kunal Kalra, Joerg Eberhard, Nona Farbehi, James J. Chong, Munira Xaymardan

**Affiliations:** ^1^Faculty of Medicine and Health, The University of Sydney, Sydney, NSW, Australia; ^2^Garvan Weizmann Centre for Cellular Genomics, Garvan Institute of Medical Research, Sydney, NSW, Australia

**Keywords:** platelet-derived growth factors (PDGF), myocardial infarction, cardiac function, matrix remodeling, angiogenesis

## Abstract

Platelet-derived growth factors (PDGFs) are powerful inducers of cellular mitosis, migration, angiogenesis, and matrix modulation that play pivotal roles in the development, homeostasis, and healing of cardiac tissues. PDGFs are key signaling molecules and important drug targets in the treatment of cardiovascular disease as multiple researchers have shown that delivery of recombinant PDGF ligands during or after myocardial infarction can reduce mortality and improve cardiac function in both rodents and porcine models. The mechanism involved cannot be easily elucidated due to the complexity of PDGF regulatory activities, crosstalk with other protein tyrosine kinase activators, and diversity of the pathological milieu. This review outlines the possible roles of PDGF ligands A and B in the healing of cardiac tissues including reduced cell death, improved vascularization, and improved extracellular matrix remodeling to improve cardiac architecture and function after acute myocardial injury. This review may highlight the use of recombinant PDGF-A and PDGF-B as a potential therapeutic modality in the treatment of cardiac injury.

## Overview of the Myocardial Infarction Pathology

Cardiovascular disease (CVD) is the leading cause of human death globally. Development of CVD depends on pathological changes in the vascular wall, including endothelium damage, intimal inflammation, vascular wall thickening by smooth muscle cell (SMC), and fibroblast activation, as well as deposition of calcium and adipose tissue in the vascular wall (Libby and Theroux, 2005). Cellular changes are mediated by humoral factors secreted by the inflammatory and activated or damaged cells, generating a milieu that supports the development of atherosclerosis and vascular pathologies. Myocardial infarction (MI) is one of the major CVD events with substantial morbidity and mortality. Following an MI, cardiomyocytes die in large numbers in the area of “ischemic attack,” and the area is repaired by fibrotic scar tissue as the postnatal cardiomyocytes in mammalians possess extremely limited regenerative capacity (Mahmoud et al., 2014).

The level of scar tissue formation and subsequent cardiac function recovery are dependent on a number of factors: (1) The size of the initial ischemic area dictated by the location of the blockage, collateral circulation, health of the microcirculation, and timing of the revascularization treatments (Bax et al., 2003). A larger area of infarct in patients with poorer collateral circulation and microvascular health (in diabetes, for example) leads to greater loss of tissue architecture (Bax et al., 2003; Adel and Nammas, 2010). (2) Infarct expansion dictated by the immune response to the injury. Overreactive immune response after MI may lead to extensive infarct expansion and worsened cardiac function outcomes (Weisman and Healy, 1987). (3) Remodeling of the scar area in accordance with extracellular matrix reconstitution and to a minor extent, if at all, cardiomyocyte regeneration (Ramos et al., 2018).

## Overview of the Effect of PDGF on Cardiac Tissue

By far, the most effective treatment for acute MI has been surgical revascularization, such as stent and bypass surgeries to restore blood supply and use of anticoagulants that prevent total occlusion of the vasculature. Stem cell treatments, especially those involving mesenchymal stem cells (MSCs), have been attempted with the intent to regenerate myocardium and improve cardiac tissue structure. Numerous animal and clinical trials have been conducted with minimally positive results in cardiomyocyte regeneration. However, improvement of cardiac function is seen in most of the trials that is thought to be attributable to the trophic benefit of the injected MSCs. The trophic effect of the MSCs alludes to the fact that cytokines or growth factors may provide similar benefits for MI without the complexity of cell delivery associated with cell culture inconsistencies and donor disparities. Indeed, many cytokines have shown their positive functions in animal models (White and Chong, 2020), but only a few were trialed in humans with the most recognized ones being the VEGF treatments (Taimeh et al., 2013; Mohl et al., 2015; Yla-Herttuala et al., 2017; White and Chong, 2020).

The authors of this review have been involved in animal research of platelet-derived growth factor (PDGF)-AB treatments in MI for many years. Overall experiences have been positive with promising outcomes seen in both rodent and porcine experiments. In a recent study by the Chong Laboratory, recombinant human (rh) PDGF-AB promoted cardiac wound repair by altering the mechanisms of scar formation of the infarcted area in a porcine model of myocardial ischemia–reperfusion. The randomized trial used 36 pigs subjected to a sham procedure or balloon occlusion of the coronary artery with a 7-day intravenous infusion of rhPDGF-AB. One-month post-MI, the survival rate of the pigs improved by 40% compared with the vehicle-treated group due to decreased ventricular arrhythmias as shown by the Holter monitor. Overall cardiac function was improved, presumably by the improved matrix configuration in the infarct area. This study provides insights into the potential clinical application of rhPDGF-AB as an adjunct to current MI treatments (Thavapalachandran et al., 2020).

This observation is supported by recent studies in rodents by our group using systemic delivery of PDGF-AB via jugular catheter connected to a minipump to the myocardial infarcted mice, which has shown improvements in cardiac anatomy and function similar to the porcine study. In addition, echocardiograms have shown a reduction in end-systolic and diastolic dimensions and scar size (Asli et al., 2017). PDGF deliveries using methods of direct intramyocardial injection, slow deliveries using nanofiber or fibrin gel implantation have also produced improved cardiac repair with differences in ligand isotypes and their delivery approaches producing diverse outcomes (Xaymardan et al., 2004; Zheng et al., 2004; Hsieh et al., 2006a; Awada et al., 2015; Table 1).

**TABLE 1:** Publications reporting exogenous platelet-derived growth factor (PDGF)/platelet-derived growth factor receptor (PDGFR) recombinant protein or antibody treatment in cardiac injury.

**Treatment types**	**Model and species**	**Outcome**
PDGF-AB combined with VEGF and angiopoietin-2 (Xaymardan et al., 2004)	Rat acute myocardial infarction (MI); intramyocardial injection	Improved cardiac function; increased survival of ear–heart transplant
PDGF-AB combined with BMC (Xaymardan et al., 2004)	Rat acute MI; intramyocardial injection	Improved cardiac function; increased survival of transplanted bone marrow cells
PDGF-AB (Thavapalachandran et al., 2020)	Porcine infarct reperfusion; intravenous continuous delivery via minipump for 7 days	Improved mortality and cardiac function; vascularity and matrix architecture
PDGF-AB (Asli et al., 2019)	Mice cute infarct intravenous continuous delivery via minipump for 5 days	Improved angiogenesis, reduced scar size, and cardiac function
PDGF-BB (Hsieh et al., 2006a)	Rat MI; self-assembling peptide nanofibers bearing ligand, sustained release for 14 days	Improved mortality and cardiac function
PDGF-A and PDGF-D (Zhao et al., 2011)	Rat with left ventricular anterior transmural MI via ligation of left coronary artery	Enhanced PDGF-A and D during angiogenesis, inflammatory and fibrogenesis response
VEGF and PDGF (Awada et al., 2015)	Myocardial injection of factors in fibrin gel	Improvement in cardiac function, ventricular wall thickness, angiogenesis, cardiac muscle survival
Neutralizing antibodies to PDGFRα and PDGFRβ (Zymek et al., 2006)	Mouse model of ischemic reperfusion; daily injection of anti-PDGFRα and anti-PDGFRβ antibodies	Anti-PDGFRα caused reduction in collagen deposition; anti-PDGFRβ caused reduction of angiogenesis

In addition to cardiac treatments, PDGF ligands have been investigated for their potential role in bone fracture healing in osteoporotic or diabetic animals (Al-Zube et al., 2009; Graham et al., 2009). Exogenous PDGF-BB has been used to treat non-healing ulcers in humans (Mustoe et al., 1994; Pierce et al., 1995). Topical application of PDGF-BB was reported to accelerate the rate of wound healing in both normal and ischemic full-thickness skin wounds, as well as burn wounds (Travis et al., 2014; Gowda et al., 2015), shortening the duration of wound healing with reduced wound contraction (Ehrlich and Freedman, 2002). Improved alveolar bone and periodontal ligament regeneration was possible when used on the patients with periodontitis as reviewed (Khoshkam et al., 2015). The applications also include worrying aspects that the PDGF and other growth factors are increasingly being used in the unregulated areas of cosmetics as wrinkle reduction measure without safety assessments (no peer data available).

PDGF ligands and receptors are essential for revascularization and stromal cell activation required for wound healing. Conversely, as mitogens, potent stimulators of mesenchymal cell angiogenesis, the PDGF/Rs are implicated in many pathological processes such as atherosclerosis, fibrosis, and tumorigenesis (Hoch and Soriano, 2003; Ostman, 2004; Olson and Soriano, 2009; Kong et al., 2014; He et al., 2015; Appiah-Kubi et al., 2016; Wang et al., 2016; Klinkhammer et al., 2018; Bottrell et al., 2019; Roehlen et al., 2020; Table 2). Search involving PDGF receptors and cardiac diseases rendered 68 types of cardiac diseases associated with PDGFRα and 106 types with associated PDGFRβ^1^. The dichotomy calls for controlled studies on the pharmacodynamic, long-term effect of the ligands before clinical application is implemented.

**TABLE 2:** Examples of publications reporting effects of endogenous/transgenic PDGF/PDGFR overexpression.

**Treatment types**	**Model and species**	**Outcome**
*Pdgfa* and *Pdgfb* overexpression (Gallini et al., 2016a)	Transgenic insertion of PDGFs under α-myosin heavy chain (α-MHC) promoter in embryos	*Pdgfa* resulted in severe fibrosis, increase in cardiac size leading to lethal cardiac failure soon after birth. *Pdgfb* led to focal fibrosis and moderate cardiac hypertrophy
*Pdgfa*, *Pdgfb*, *Pdgfc* and, *Pdgfd* overexpression (Gallini et al., 2016a)	Intramyocardial delivery of *Pdgf isoforms* using adenoviral vector in adult mice	Varied reactions observed: *Pdgfa* and c resulting in small scars, while *Pdgfb* results in extensive scarring
PDGF-C overexpression (Ponten et al., 2003)	Transgenic insertion of PDGF-C under α-MHC promoter in embryonic and adult mice	Cardiac fibroblast proliferation, deposition of collagen, hypertrophy, vascular defects, and the presence of Anitschkow cells in the adult myocardium
PDGF-D overexpression (Ponten et al., 2005)	Transgenic insertion under α-MHC promoter	Dilated cardiomyopathy and subsequent cardiac failure

In this review, we attempt to elucidate the roles of PDGF signaling in the prevention of cell death, improvement of vascularity, and a potential role in myocardial regeneration as well as matrix remodeling. The focus is on the PDGF-A and B ligands and their receptors α and β in cardiac repair. Aberrant signaling of PDGF in pathological conditions is also covered although to a lesser extent due the scope of this review.

## Role of Platelet-Derived Growth Factor in Cardiac Injury Models

### Platelet-Derived Growth Factor and Platelet-Derived Growth Factor Receptors

PDGFs are a group of multifunctional proteins that play key roles in the processes of embryonic development, organogenesis, and formation of blood vessels (Betsholtz, 1995; Hoch and Soriano, 2003). PDGF consists of four polypeptide chains, namely, the PDGF-A, PDGF-B, PDGF-C, and PDGF-D, which form four homodimers including PDGF-AA, PDGF-BB, PDGF-CC, and PDGF-DD and one heterodimer, PDGF-AB (Li et al., 2000; Bergsten et al., 2001; Kazlauskas, 2017; Yue et al., 2019).

**FIGURE 1:**
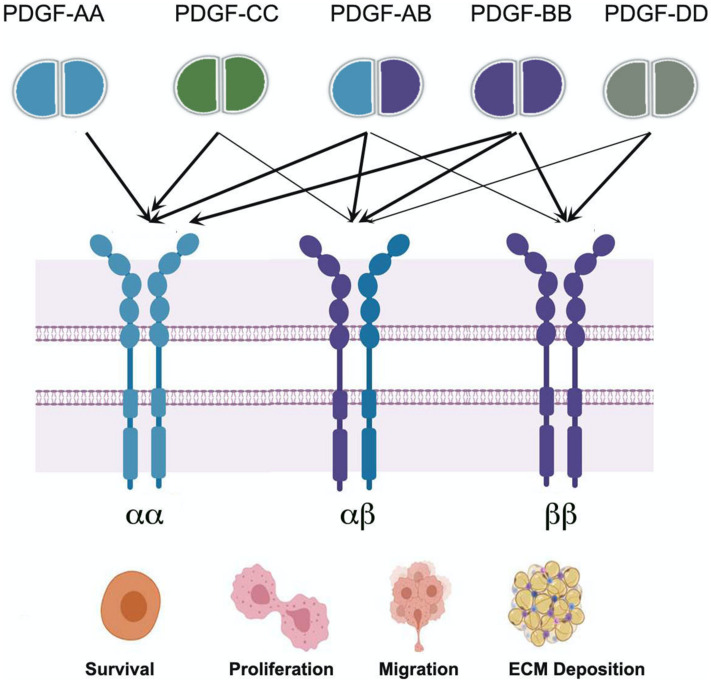
Schematic depiction of platelet-derived growth factor (PDGF)/platelet-derived growth factor receptor (PDGFR) types, interactions and ligand functions in cellular survival, proliferation, migration, and ECM deposition. The lighter arrows represent weaker binding of ligand to the receptor, while the darker arrows show high-affinity receptor binding (Gallini et al., 2016b). This sketch was generated with BioRender.com.

These PDGF ligands exert their functions by binding to two types of receptors (Figure 1) that are usually localized to connective tissue cells. PDGFα and β belong to class III receptor tyrosine kinase (RTK) and have different expression patterns and physiological roles. The extracellular region of the receptor consists of five immunoglobulin-like domains that bind to ligands, while the intracellular part is a tyrosine kinase domain for downstream transduction. Downstream signal transduction pathways include phosphatidylinositol 2 kinase, MAPK, PI3K, Src family kinases, and phospholipase Cγ (Valius and Kazlauskas, 1993; Chiariello et al., 2001).

The PDGF ligands help maintain homeostasis and remain relatively dormant and tightly controlled in adult tissues with transient enhancement of expression occurring during wound healing. Dysregulation of these processes leads to pathologies such as fibrosis and cancer (Ostman, 2004; Olson and Soriano, 2009).

PDGF ligands are extremely stable molecules even in 100°C heat and varied pH conditions (Antoniades et al., 1979) and, thus, presumably suitable for sustained delivery.

#### PDGFRα

PDGFRα is expressed by widely distributed non-vascular interstitial fibroblasts (Ivey and Tallquist, 2016; Sebastiao et al., 2018) including a subpopulation of cells marking enriched MSC populations in all organs/tissues (Farahani and Xaymardan, 2015; Ivey and Tallquist, 2016). Detailed single-cell RNA (scRNA) analysis has shown that while the PDGFRα cells in the mouse hearts are mostly fibroblastic in nature, a small population co-expresses endothelial marker CD31, and a further minor population co-expresses macrophage markers. Both CD31 and macrophage marker expressions in PDGFRα cells are upregulated in post-MI hearts at day 3 and day 7 (Farbehi et al., 2019), although it is unclear whether this is due to transdifferentiation or an influx of cells from elsewhere (Souders et al., 2009). It is accepted that cardiac PDGFRα cells are derived either from the mesoderm via the proepicardial organ (Soriano, 1997; Chong et al., 2011; Smith et al., 2011) or the neural crest (Tallquist and Soriano, 2003). During homeostasis, they play a supportive role to the parenchymal cells of the tissues and stay relatively dormant. In pathology, PDGFRα signaling including reactivation of epicardium promotes angiogenesis and extracellular remodeling to restore tissue integrity and tensile strength (Horikawa et al., 2015; Quijada et al., 2020).

**FIGURE 2:**
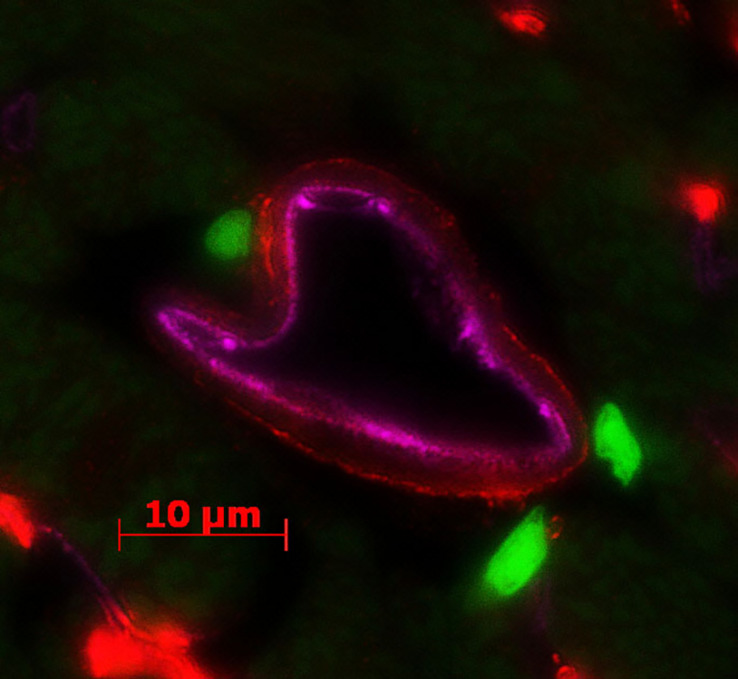
PDGFRα and PDGFRβ expression in the healthy cardiac tissue of a *Pdgfr*α*^*GFP*^* mouse. PDGFRα^+^ (green, *Pdgfr*α*^*GFP*^*) cells are located in the area of non-vascular interstitium, while PDGFRβ (red, antibody staining against PDGFRβ) is expressed in the pericytes surrounding the endothelium (pink, immunostaining of CD31).

In mouse hearts, over 70% of the PDGFRα cells are positive for SCA1 (Houlihan et al., 2012), which contains a rare population of cardiac CFU-F-forming cells or so-called MSCs, while the SCA1 negative population does not give rise to CFU-F (Chong et al., 2011). These cells migrate from the epicardial organ at E9.5 in mouse embryos and reside in the type VI collagen matrix outside of the vascular structure (Munoz-Chapuli et al., 2002; Chong et al., 2011). They are negative for, or low in, PDGFRβ and other pericyte markers (Santini et al., 2020; Figure 2). *Pdgfr*α is also expressed in the cardiomyogenic progenitors in the lateral plate mesoderm in embryos prior to heart tube formation (Bloomekatz et al., 2017; Yoon et al., 2018), later diminishing with only a small population of NKX2.5-positive myocytes co-expressing PDGFRα in the right atrial area in embryonic day 8.5 in mice (Prall et al., 2007). PDGFRα-positive cardiomyocyte progenitors may exist in small numbers in adult human hearts, and whether or not this can contribute to the repair of myocardium following injury is unknown (Chong et al., 2013). In embryos, *patch* deletion of *Pdgfr*α leads to cardiac defects, including enlarged hearts and septum defects, as well as epicardial malformation, resulting in early embryonic lethality in mice (Orr-Urtreger et al., 1992; Soriano, 1997; Bax et al., 2010). In adult injury models, PPDGFRα cells are thought to mainly give rise to myofibroblasts and lipofibroblasts as reported in models of pulmonary injury (Li et al., 2018) and myofibroblast in cardiac injury model (Asli et al., 2019). The differentiated myofibroblasts express a lower level of PDGFRα tested by scRNA or flow cytometer, respectively.

#### PDGFRβ

PDGFRβ is expressed in the cardiac pericytes, which are predominantly found at vascular locations (Figure 2). The expression and phosphorylation of *Pdgfr*β in cardiomyocytes increase dramatically in response to pressure overload stress (Chintalgattu et al., 2010; Yue et al., 2019). *Pdgfr*β knockout produces embryonic lethality due to hemorrhage from vascular malformation due to impaired pericytes and/or defects in hemopoiesis. Inactivation of PDGFRβ signaling leads to cardiac abnormalities, including ventricular septal defects, late embryonic ventricular dilation, lack of coronary vascular SMCs, myocardial hypertrabeculation, and hemorrhage (Hellstrom et al., 1999; Bjarnegard et al., 2004; Mellgren et al., 2008). Cardiomyocyte-specific inducible deletion of *Pdgfr*β (*Pdgfr*β*^*Nkxcre*^*) in embryos, however, do not exhibit remarkable malformation, but when deleted in adults, the mice develop severe ventricular dilation and heart failure in response to pressure overload with a possible mechanism of impaired activation of Akt and MAPK pathways (Chintalgattu et al., 2010). In recent scRNA studies in adult injuries, PDGFRβ is found in circulating fibrocytes and myofibroblasts in kidney fibrosis and myofibroblasts in lung injury models (Kramann et al., 2018; Li et al., 2018).

#### PDGF-A

Protein is encoded by the PDGF-A gene, which encodes for PDGF-A and VEGF. PDGF-A is proteolytically cleaved to form a subunit and either homodimerize or heterodimerize with the B subunit. PDGF-AA is widely expressed in the embryonic and postnatal tissues including the heart with knockout producing embryonic lethality (Andrae et al., 2014). PDGF-AA acts mostly in a paracrine signaling manner post-gastrulation, interacting with PDGFRα in embryos where the ligand is expressed in endoderm or ectoderm and the receptor predominantly in mesoderm (Bloomekatz et al., 2017). In adults, the ligand is frequently found in the epithelium or endothelium. In the normal adult heart, PDGF-A is shown to be expressed in the interstitial cells where the receptor-positive cells reside, and the level of PDGF-A is significantly increased following MI (Zhao et al., 2011).

#### PDGF-B

Protein is encoded and proteolytically cleaved similar to the A subunit. This ligand is the only PDGF that binds to all three receptor combinations with high affinity and is required for normal proliferation and recruitment of pericytes and vascular SMCs. Knockout embryos develop septum defects, underdeveloped valvular structure, abnormal cardiac innervation, and hypoplastic compact myocardium largely similar to cases of *Pdgfr*β deletion; *Pdgfr*β knockout embryos also die from hemorrhage due to the lack of pericyte lining of the blood vessels (Leveen et al., 1994; Van den Akker et al., 2008).

#### *Pdgfa/r*α and *Pdgfb/r*β Signaling May Have Distinct Functions in Development and Adult Homeostasis and Pathology

It is believed that *Pdgfa/r*α and *Pdgfb/r*β can cross-activate each other and may have overlapping functions. However, the “overlap” is perhaps partial at best, as mutation of one fails to be compensated by the other during development. Deletion of *Pdgfa/r*α and *Pdgfb/r*β present non-identical developmental malformations. This evidence indicates a lineage independence of *Pdgfa/Pdgfr*α and *Pdgfb/Pdgfr*β signaling. The lack of compensatory offset also indicates that both autocrine and paracrine signaling processes are important in this ligand/receptor interaction. For example, autocrine signaling activation is necessary in early embryos or in pathological states where more homogenous tissue expansion is prioritized, and paracrine signaling may come into play for the organized interactions between diverse cell types to aid in migration and differentiation (Palmieri et al., 1992).

**FIGURE 3:**
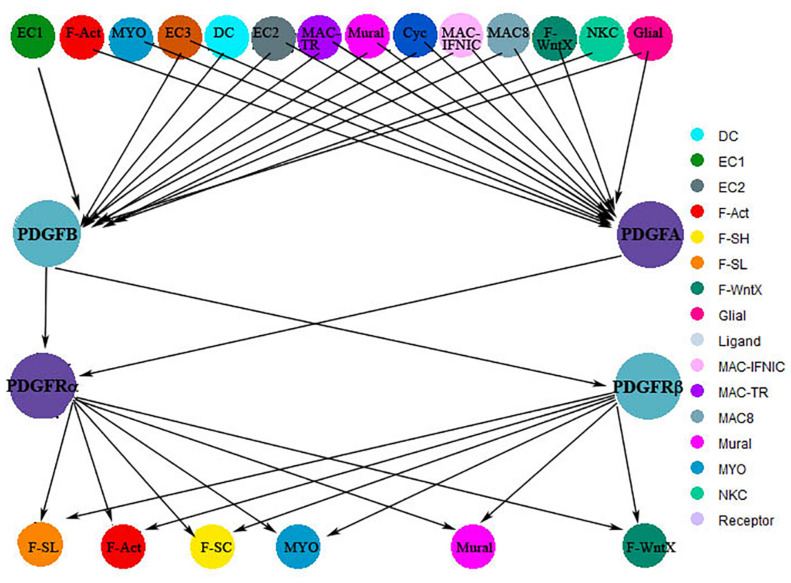
Tree plot showing signaling networks of PDGF-A and B ligands and receptors α and β at day 7 post-MI. Cardiac interstitial cells shown at the top layer of this graph secrete PDGF-A/B, which interacts directly with the α and β receptor-presenting cells shown at the bottom layer. These data was derived from a publication by Farbehi et al. (2019). The analysis did not show significant changes in C and D ligands post-MI in this model.

Upregulation of both receptors and PDGF-B is noted at day 7 in a mouse ischemic reperfusion model, with blockage of the PDGFRβ causing leaky blood vessels, while blocking the PDGFRα significantly decreases collagen deposition in the infarct, both impairing healing (Zymek et al., 2006). Single-cell analysis at day-7 post-MI has shown involvement of the interstitial cells in PDGF ligand secretion, the cell types including endothelial, fibroblast, immune, and mural cells. The interaction of the ligands with their own receptors and receptors that mediate various cellular activities is illustrated in Figure 3, demonstrating the targeting of interstitial cell types that contribute to angiogenesis, collagen deposition, cell proliferation, and mural cell regulations (Farbehi et al., 2019; Figure 3).

#### PDGF-C and D

PDGF-C and D are synthesized and secreted as latent growth factors, which require proteolytic removal of the N-terminal CUB domain for receptor binding. The C and D ligands only form homodimers. C and D ligands work through the dimerized receptors α and β similar to ligands A and B. However, they differ from A and B ligands in their molecular structures with longer pro-sequences that include a large N-terminal CUB domain, and the relative hydrophilicity of these ligands may make them less flexible and have shorter binding duration for receptors compared with A and B ligands, which in turn produce a differed effect of the PDGF signaling cascade, as reviewed (Fredriksson et al., 2004; Chen et al., 2013). *Pdgfc*^–/–^ mice display cleft palate and craniofacial malformation (Ding et al., 2000), while *Pdgfd*^–/–^ mice present mild vascular abnormality and disorganized NG2^+^ pericytes, their offspring are born generally healthy and enjoy a normal lifespan (Gladh et al., 2016).

## Roles in Anti-Apoptosis

Reducing myocardial cell death following an ischemic insult is desirable and is currently the most effective treatment available in developed countries where most patients receive percutaneous angioplasties to reduce the duration of ischemia and prevent further cell death (Castellani et al., 2010) but not without complications (Hausenloy and Yellon, 2013; Davidson et al., 2019).

PDGF is a principal survival factor that inhibits apoptosis and promotes proliferation (Harrington et al., 1994). As the mechanisms of cell proliferation and differentiation are intrinsically linked to the process of apoptosis, the default of proliferating cells is to die unless specific survival signals are provided (Harrington et al., 1994). PDGF roles in anti-apoptosis have been linked to activation of cMyc and Ras/PIK13 pathways (Romashkova and Makarov, 1999) in cancer cells and indeed cardiomyocytes (Hsieh et al., 2006a).

In rodents, delivery of PDGF-AB via direct myocardial injection 24 h prior to MI or co-injection with VEGF and angiopoietin-2 at the time of coronary occlusion was able to suppress acute myocardial cell death by >50%, thereby reducing the extent of MI by providing a cardioprotective benefit (Xaymardan et al., 2004).

PDGF-BB was tested on the resistance of apoptotic induction in engineered tissue from neonatal rat cardiomyocytes. The treated hearts were at least partially protected from caspase-3-induced apoptosis (Vantler et al., 2010). Peptide nanofibers (NF) with PDGF-BB injected into the myocardium ensured a sustained release of the PDGF-BB after coronary occlusion in the rats, which dramatically decreased caspase-3 activation after 1 day, reducing apoptosis in a dose-dependent manner. It was demonstrated that the activation of Akt in the myocardium is induced by injection of NF/PDGF-BB, showing that this strategy induces survival signaling in the myocardium *in vivo* (Hsieh et al., 2006a).

Impaired PDGFRα pathways lead to apoptosis in relevant tissues. *Pdgfr*α deletion in embryos resulted in apoptosis in the receptor-expressing tissue, contributing to malformation of the embryos (Qian et al., 2017). Similarly, conditional deletion of *Pdgfr*α caused mesenchyme apoptosis and urorectal developmental anomalies in mice (Qian et al., 2019).

PDGFRβ signaling is important for cardioprotection as shown in the aortic constriction model where PDGFRβ was upregulated and phosphorylated in response to stress. Conditional knockout of *Pdgfr*β (*Pdgfr*β*^*Nkx*2.5*cre*^*) leads to ventricular dilation with age and severe heart failure upon induction of pressure overload. AKT/ERB signaling pathways were also indicted in these experiments (Chintalgattu et al., 2010). The protective effect of the PDGF may be dose dependent as shown in cultured vascular MSCs; the increased dose of PDGF-BB induced apoptosis via *bcl2* upregulation or inactivation of BAD (Okura et al., 1998; Zhou et al., 2000).

Overall, the anti-apoptotic properties of the PDGFRα and PDGFRβ are reasonably clear. While the ligands and receptors play a certain role in earlier embryonic cardiac development, the mechanism may largely be dependent on epi/endocardial induction. The cardiomyocyte expression of these factors cannot be entirely ruled out and may be minimal. PDGF-B and PDGFRβ signaling, on the other hand, seems to be more profoundly involved in the anti-apoptotic function of these factors, achieving cardioprotection through a specific downstream signaling pathway dictated by AKT/ERB.

## Role in Targeting Angiogenesis

The majority of cardiac disorders stem from vascular dysfunction and poor perfusion. The problem further deteriorates after MI, where cardiac hypertrophy and fibrosis cause a further decrease in vascularity to cellularity ratio, worsening perfusion deficit of the cardiac tissue. Encouraging angiogenesis pre- or post-MI will help protect cardiomyocytes from further apoptosis and improve the microenvironment for cellular functions of the heart.

PDGF/R are well-accepted vasculogenic and angiogenic promoters by either direct participation in vascular assembly by α/β receptor-presenting cells or by providing migratory cytokines and extracellular matrix support for vessel formation. *Pdgfr*α-positive cells contribute early embryonic endothelial cells in the lateral plate mesoderm (Ding et al., 2013). In adults, *Cre*-induced *Pdgfr*α knockout suppressed neovascularized areas in implanted sponge presumably through suppression of the wound matrix assembly (Horikawa et al., 2015). PDGFRβ is involved in an assembly of principle vascular cell types, the endothelium, SMA, and pericytes to form blood vessels in many organs of embryos (Hellstrom et al., 1999). PDGF-BB is a major mitogen for vascular endothelial cells and are involved in pericyte recruitment, which also can directly stimulate endothelial cell proliferation *in vitro* as well as in embryonic chorioallantoic (Battegay et al., 1994; De Marchis et al., 2002) and vascular growth in hind limb ischemia model in adult mice (Brown et al., 1995). Injection of PDGF-BB into rabbit myocardium was shown to indirectly stimulate angiogenesis by increasing VEGF protein production (Affleck et al., 2002). Conversely, *Pdgfb*-deficient mice endothelium unable to recruit pericytes, thus, form microaneurysm (Lindahl et al., 1997).

Both PDGFRα and β are expressed in the culture endothelial cells (Lee et al., 2013) and newly formed blood vessels in mouse cornea (Cao et al., 2002). Minor populations of cardiac endothelial cells express PDGFRα with an increase in CD31^+^PDGFRα^+^ cells seen post-MI, indicating that the PDGFRα is conducive to post-MI angiogenic regulation during cardiac wound healing (Zhao et al., 2011; Awada et al., 2015; Farbehi et al., 2019). PDGF ligands can directly activate both endothelial cells and pericytes. It is also possible that family cross-family PDGF to VEGFR2 interactions exist, for example. VEGF-A is proposed to bind directly to PDGFRα and β; Conversely, PDGFs crosstalk to VEGF receptors to enact angiogenic function (Mamer et al., 2020).

In MI models, promoting vascularity through both AB and BB ligand delivery has been shown to improve the post-MI milieu and cardiac function, although questions on long-term benefits and effectiveness in humans are still unclear. PDGF-AB (Xaymardan et al., 2004) and BB (Hsieh et al., 2006a) delivery directly into the myocardium in mouse and rat models and systemic delivery in mouse and pig models have all shown improvements in angiogenesis in post-MI cardiac tissue, which is presumably, in part, responsible for improved cardiac function and myocardial anatomy. In these studies, the roles of PDGF-A and B are not dissected. PDGF-AB seems to generate stable vessels and arterioles (Zhang et al., 2009). PDGF-C, as a newer ligand in the PDGF family, has been increasingly shown to induce angiogenesis via PDGFRα (Cao et al., 2002; Lee and Li, 2018), however, its role has not been investigated from the perspective of cardioprotection.

No pharmacodynamic studies have been performed; however, sustained delivery methods such as intravenous infusions (Chong et al., 2011) and sustained release using nanoparticles (Hsieh et al., 2006a) or fibrin gel (Su et al., 2020) may be superior to one-off direct injections into the myocardium. In the case of one-off injection, concomitant delivery of other factors (e.g., VEGF) may be necessary. The newer PDGF ligand C has been increasingly associated with angiogenesis.

## Targeting Cardiomyocyte Proliferation

The ideal scenario after a cardiac injury would be the regeneration of cardiomyocytes to restore contractile function of the heart, ensuring normal cardiac output. However, rebuilding the myocardium through regeneration of the cardiomyocytes in the adult mammalian seems to be a formidable task. The difficulty is highlighted by the collapse of the “house of cards” built by Anversa et al. around the notion of using bone marrow or cardiac cKit cells to regenerate cardiomyocytes (Davis, 2019).

In reality, carbon-14 dating results indicate that adult cardiomyocytes may have a low-rate cell cycling ability (Bergmann et al., 2009) that is far from sufficient for the heart to recover from a catastrophic insult such as MI. Zebrafish and some amphibians, even neonatal mice, display regrowth of cardiomyocytes predominantly via cell cycle reentry mechanisms (Wills et al., 2008; Porrello et al., 2011), but adult mammalian hearts do not seem to possess this advantage.

Whether or not PDGF can stimulate regeneration of cardiomyocytes is elusive. However, it is clear that PDGFs and their receptors play important roles in the early development during the heart tube formation stage when primordial cardiac crescent myocytes fuse along the midline. PDGF is secreted by the adjacent endoderm to guide the migration and fusion of the heart tube with the mutation of *Pdgfr*α disrupting the heart tube assembly in both zebrafish and mice (Bloomekatz et al., 2017). Initial embryonic heart tissue is founded by non-dividing cardiomyocyte progenitors; the proliferation resumes following cardiac looping, especially in the compact myocardium. Cardiomyocyte proliferation is induced by epicardial-derived signals following the establishment of cardiac fibroblasts marked by PDGFRα and predominantly derived from the epicardium (Chong et al., 2011); this indicates the mitotic function of PDGFRα signaling.

As determined by measurements of the atrioventricular length and left ventricular length and width, exposure of rat embryos to PDGF-AA resulted in a 42% increase in total protein levels in the heart but did not result in a significant increase in heart growth. Exposure of embryos to PDGF-BB resulted in a 77% increase in total protein levels and a significant increase in the measured heart parameters. Although a comparison of control and PDGF-AA-treated embryos showed no increase in the overall size of the heart, confocal microscopy showed an increase in the size and number of myofibrillar bundles in the developing myocardium (Price et al., 2003). No cardiomyocyte proliferation assay was performed; thus, the observation of increased heart size stimulated by PDGF was likely due to hypertrophic response of the cardiomyocytes and interstitial cell proliferation.

Intravenous infusion of PDGF-AB in mice with surgical MI has been shown to increase EDU-positive myocyte numbers in the remote area of the sub-endocardium. However, it is unclear whether the DNA activities are due to cytokinesis or karyokinesis (Asli et al., 2017). The mechanism behind this observation is not clear but mostly likely to be attributed to the paracrine effect of the interstitial cell activation rather than a direct effect.

PDGFRα may demark cardiomyocyte progenitors in mouse and human embryonic stem cells (Uosaki et al., 2012). A rare population of cardiomyocytes express PDGFRα in human fetal and adult hearts, and a small fraction of PDGFRα cells are able to differentiate into cardiomyocytes *in vitro* (Chong et al., 2013) notwithstanding that the *in vivo* effect of these cells in injury repair is unknown. Recently, Yue et al. (2019) reported that with age, levels of phospho-PDGFRβ decreased, and this correlated to the loss of cardiomyocyte proliferative capacity after apical resection. For example, high expression of phospho-PDGFRβ was seen in mice up to 7-day postnatal age, and only trace amounts were retained in the adult mice hearts. Cardiac-specific and sustained activation of the PDGFRβ restore cardiomyocyte regenerative capacity in adult mice. This proliferation was driven by an upregulation of the enhancer of ZESTE homolog2 EZH2, which promotes the proliferation of cardiomyocytes with the conditional knockout of Ezh2 blocking CM proliferation (Yue et al., 2019), although genetic upregulation of the PDGF pathways may risk extensive fibrosis after injury as discussed below.

## Platelet-Derived Growth Factor in Matrix Remodeling/Wound Strength/Anti-Arrhythmia: Targeting Fibroblasts

Perhaps the most authentic and prominent roles of the PDGFs are in the areas of improving matrix remodeling and tissue architecture configuration. There is clear evidence that the *Pdgfr*α is expressed in most of the mesenchymal cell population, including the post-gastrulation mesoderm/neural crest stem cells, progenitors of bone, teeth, and fibroblasts. These are all matrix producers that provide structural and humoral support to parenchymal cells (not excluding the potential of their own differentiation to parenchymal cell types such as osteocytes and odontoblasts) (Farahani and Xaymardan, 2015). Similarly, *Pdgfr*β is a prominent pericyte marker, participating in the maintenance of vascular bed and extracellular matrix remodeling.

In adults, PDGF-AA or PDGF-AB induce fibroblast proliferation (Lepisto et al., 1995), and PDGF-BB is a potent recruiter of the pericytes and SMA. PDGF-BB has also been shown to alter matrix integrin expressions, which may alter the chemotaxis of the fibroblasts in ECM, therefore, changing the ECM properties (Xu and Clark, 1996). PDGF-D has been shown to increase fibroblast activity in rat hearts (Zhao et al., 2013).

The mechanistic pathway by which the PDGFs stimulate ECM deposition may be in collaboration with TGF-β through both non-*Smad* and *Smad*-dependent manners (Fischer et al., 2007). Activation of MAPK and PI3K have been shown to modulate the extracellular matrix composition and stiffness via Wnt/β-catenin (Astudillo, 2020). PDGFRα is required for TGFβ signaling of cultured human hepatic satellite cell transdifferentiation to myofibroblasts, and PDGFRα knockdown inhibits *Smad*-dependent TGFβ signaling (Liu et al., 2014). Activities of PDGF efficiently are suppressed by TGFβ neutralization or interference with the SMAD/TβR1 or PI3K/Akt pathway (Charbonneau et al., 2016). The complexity is not easily dissected but indicative of mutual regulatory effect between these potent ECM modulators.

In the myocardium, interstitial non-cardiomyocyte cells entangled within a network of extracellular matrix proteins providing structural support, network communication, and humoral signaling to the organ, acting as the primary “caretakers” of the health of the extracellular matrix. In acute diseases such as MI, the cardiac fibroblasts rapidly proliferate and change in phenotype and function (e.g., from negligible expression of SMA to SMA-rich myofibroblasts), and deposit extracellular matrix to prevent cardiac rupture in mammalians. The types of extracellular matrix deposition may have importance in cardiac tissue elasticity and, therefore, cardiac function recovery. Stiffness and elasticity may also impact the stem/progenitor functions and have further impact on the regeneration of cardiomyocytes and endothelium alike. Studies show that increased stiffness has detrimental effects on cardiac recovery, as it suppresses early cardiac marker expression (Ranganath et al., 2012; Sullivan et al., 2014). Crosstalk of the fibroblasts with cardiomyocytes may also modulate cardiomyocyte proliferation or hypertrophy depending on the fibroblast phenotype (Musa et al., 2013). Utilizing the natural reparative processes of fibroblasts to modify properties of the forming cardiac scar is quietly emerging as an exciting therapeutic avenue.

General consensus regarding the role of PDGF ligands and receptors is that they are profibrotic, and gain of function studies largely support this notion.

Overexpression, or persistent expression of PDGFs or their receptors have serious adverse effect specifically on the heart. For example, the transgenic expression of the natural isoforms of *Pdgfa* and *b* under the α-myosin heavy chain (α-MHC) promoter has shown severe fibrotic reactions, increase in cardiac size, and cardiac failure a week after birth, whereas overexpression of *Pdgfb* showed focal fibrosis and cardiac hypertrophy (Gallini et al., 2016b).

Cardiac-specific overexpression of PDGF-C conditional to α-MHC increased cardiac fibroblast proliferation, collagen deposition, hypertrophy, and vascular defects and, additionally, sex-dependent changes, such as male mice exhibiting hypertrophy and female mice experiencing dilated cardiomyopathy and heart failure (Ponten et al., 2003). Transgenic overexpression of PDGF-D stimulated the proliferation of cardiac interstitial fibroblasts and arterial vascular SMCs, resulting in cardiac fibrosis followed by dilated cardiomyopathy and subsequent cardiac failure (Ponten et al., 2005). Expression induced by intramyocardial adenoviral-mediated delivery; however, produced slightly mixed results showing that *Pdgfc* and *c* decreased the amount of scar tissue and increased the numbers of PDGFRα-positive fibroblasts, while *Pdgfb* induced large scars with extensive inflammation, and *Pdgfd* produced a small and dense scar (Gallini et al., 2016a).

A recent study on cardiomyocytes differentiated from induced pluripotent stem cells (IPSCs) was derived from an LMNA mutant patient showing single-cell base arrhythmia. The authors found an increase in PDGFRβ in these mutant cardiomyocytes. Interestingly, they found that inhibition of the PDGF signaling pathway ameliorated the arrhythmic *in vitro* (Lee et al., 2019).

The recent preclinical porcine study exploring the role of PDGF-AB in the attenuation of myocardial fiber heterogeneity within a scar and organized collagen fiber alignment have shown significant improvements in cardiac function and a reduction in cardiac arrhythmia. Musa et al. (2013) showed that the atrial myocytes demonstrated significant disturbance in calcium channel density to potentially provoke arrhythmia, which can be neutralized when myofibroblasts are pretreated with PDGF-AB.

The role of exogenously delivered ligands in fibrosis is less clear. Types of ligands, dosage, and duration of ligand presence, as well as activated cell types may switch the processes from a protective would healing and tissue regeneration to adverse effect of fibrosis or tumor formation.

Santini et al. (2020) demonstrated the diversity in the *Pdgfr*α cell cultured from injured and uninjured mice skeletal muscle tissues. The study showed that the activated and terminally differentiated *Pdgfr*α cells lose their regenerative capacity and display fibrotic activities. A subpopulation of Gli-1-positive PDGFRβ cells was more likely to differentiate into myofibroblasts (Kramann et al., 2015) indicating that inhibition or promotion of a specific subpopulation may avoid widespread adverse effect when using the PDGF pathway as a treatment modality.

## Conclusion and Future Perspective

Preclinical trials in porcine model have shown promising evidence that PDGF-AB exerts a cardioprotective effect following MI with rodent studies reporting similar results (Xaymardan et al., 2004; Zheng et al., 2004; Hsieh et al., 2006a,b; Awada et al., 2015; Asli et al., 2017; Thavapalachandran et al., 2020). Although no human trials have been performed thus far, the results from animal studies suggest a possible clinical application of PDGF ligands in improving therapeutic outcomes in MI patients. Short-term treatments used in swine and rodent studies showed no adverse effects of PDGF-AB when delivered intravenously for 7 days. In addition, the matrix remodeling seen in the porcine model points to a favorable tensile strength augmentation that contributes to improved cardiac function and reduced arrhythmia, which was thought to contribute to the reduced mortality in the subjects. No extensive scarring was observed in either the pig or mice studies (Asli et al., 2019; Thavapalachandran et al., 2020). Intravenous infusion of PDGF-AB in mice also failed to demonstrate increased myofibroblast activity post-MI using scRNA analysis (Asli et al., 2019). Indeed, there is a clear link between PDGF signaling pathways and fibrosis. The most convincing supporting evidence is derived from genetically upregulated murine models where increased PDGFRα is shown to cause lung fibrosis and conditional knockout of PDGFRα shown to attenuate liver fibrosis (Andrae et al., 2008). However, there is no clear evidence that exogenous ligand delivery would produce similar effects. In fact, improved matrix remodeling seems to be beneficial for the healing of cardiac tissue after injury. Studies of PDGF-AB delivery in pigs resulted in directional synchrony in collagen fibers, which may have contributed to better preserved architecture of the infarcted area and to the vascularity of scar tissue (Thavapalachandran et al., 2020).

Matrix remodeling is one of the mechanisms that facilitates improved functional outcomes after infarction. PDGF pathway regulation also contributes to improved cell survival, increasing angiogenesis and, perhaps to a lesser degree, activation of the cardiomyocyte cell cycling machinery, although this pathway may not be potent enough to induce cellular proliferation and contribute to the regeneration of the myocardium. The exogenous delivery of PDGF to aged mice also compensates for age-related downregulation in the PDGF pathway (Xaymardan et al., 2004).

Inhibition of PDGF pathways, on the other hand, leads to worsened cardiac outcomes in mice after MI. For example, administering PDGF receptor inhibitor imatinib to mice experiencing MI caused a reduction in vascularity and ejection fraction when compared with non-treated mice (Fazel et al., 2006). It can be argued that the imatinib is not specific to PDGFRs as it also impacts c-Kit. However, specific neutralizing antibodies against PDGFRα and β have led to reductions in vascularity and collagen deposition in post-infarction hearts and demonstrated the importance of PDGF receptors in cardiac wound healing (Zymek et al., 2006). Interestingly, neutralizing antibodies also had effects on inflammatory cells, anti-PDGFRα resulted in increased macrophage infiltration, while the inhibition of PDGFRβ prolonged the duration of leukocyte infiltration post-MI (Zymek et al., 2006). This indicates that PDGF signaling pathways may have additional roles in immune regulation that impacts repair post-MI. This is further supported by our studies showing M1/M2 conversion in the PDGF-AB-treated mice (Asli et al., 2019). Excessive inflammation is related to fibrosis and worsened infarct expansion, which can be attenuated by anti-inflammatory treatments that produce beneficial effects both in animal models and human trials of MSC treatments, as most of the benefits seem to be attributable to the anti-inflammatory effect of the MSC trophic activities. In some cases, more potent anti-inflammatory drugs are used to treat acute MI patients with improved mortality and cardiac function (Giugliano et al., 2003), although high-dose steroid can produce opposite effects by reducing collagen deposition (Giugliano et al., 2003).

To date, the primary pharmaceutical applications of PDGFs have been the inhibition of PDGF pathways in the treatment of cancer and the prevention of fibrosis with clinically approved drugs including small inhibitory molecules, such as imatinib, and protein antagonists (Bruggemann et al., 1989; Wollin et al., 2014; Ying et al., 2017; Papadopoulos and Lennartsson, 2018; Olsen et al., 2019). Pharmacological augmentation studies regarding therapeutic roles of PDGFs in cardiac repair and overall wound healing aspects are limited. Studies on direct comparison of all five dimers of PDGFs would be valuable for identifying the most useful isotypes or the combinations of PDGFs that improve mortality and morbidity and cardiac function. Pharmacodynamics on dose, duration, and benefit versus toxicity including longer-term adverse effect will be informative for establishing applicational modalities. These studies will be formidable due to the laboriousness of the cardiac injury models and the cost of the recombinant proteins. Future studies including animal models with risk factors of hypertension and diabetes would be presentative of the human population with MI risk. The mechanism of the beneficial roles of the PDGF in cardiac healing or regeneration is not elucidated. The complexity of the pathway suggests that the PDGFs provide a multitude of benefits to improve many aspects of cardiac wound healing and may be a promising therapeutic target for the treatment of post-MI cardiac pathologies.

## Author Contributions

KK drafted the manuscript. NF drafted the figures and data analysis. JE and JC contributed to writing. MX constructed the structure and contributed to writing and proofing. All authors contributed to the article and approved the submitted version.

## Conflict of Interest

The authors declare that the research was conducted in the absence of any commercial or financial relationships that could be construed as a potential conflict of interest.

## Publisher’s Note

All claims expressed in this article are solely those of the authors and do not necessarily represent those of their affiliated organizations, or those of the publisher, the editors and the reviewers. Any product that may be evaluated in this article, or claim that may be made by its manufacturer, is not guaranteed or endorsed by the publisher.

## Footnotes

1
https://platform.opentargets.org/target/ENSG00000134853?view=sec:known_drug

## References

[B1] Adel W. Nammas W. (2010). Predictors of contractile recovery after revascularization in patients with anterior myocardial infarction who received thrombolysis. *Int. J. Angiol.* 19 e78–e82. 22477594 10.1055/s-0031-1278373 PMC3005410

[B2] Affleck D. G. Bull D. A. Bailey S. H. Albanil A. Connors R. Stringham J. C. (2002). PDGF(BB) increases myocardial production of VEGF: shift in VEGF mRNA splice variants after direct injection of bFGF, PDGF(BB), and PDGF(AB). *J. Surg. Res.* 107 203–209. 10.1006/jsre.2002.6510 12429176

[B3] Al-Zube L. Breitbart E. A. O’Connor J. P. Parsons J. R. Bradica G. Hart C. E. (2009). Recombinant human platelet-derived growth factor BB (rhPDGF-BB) and beta-tricalcium phosphate/collagen matrix enhance fracture healing in a diabetic rat model. *J. Orthop. Res.* 27 1074–1081. 10.1002/jor.20842 19170096

[B4] Andrae J. Gallini R. Betsholtz C. (2008). Role of platelet-derived growth factors in physiology and medicine. *Genes Dev.* 22 1276–1312. 10.1101/gad.1653708 18483217 PMC2732412

[B5] Andrae J. Gouveia L. He L. Betsholtz C. (2014). Characterization of platelet-derived growth factor-A expression in mouse tissues using a lacZ knock-in approach. *PLoS One* 9:e105477. 10.1371/journal.pone.0105477 25166724 PMC4148317

[B6] Antoniades H. N. Scher C. D. Stiles C. D. (1979). Purification of human platelet-derived growth factor. *Proc. Natl. Acad. Sci. U.S.A.* 76 1809–1813. 287022 10.1073/pnas.76.4.1809 PMC383481

[B7] Appiah-Kubi K. Wang Y. Qian H. Wu M. Yao X. Wu Y. (2016). Platelet-derived growth factor receptor/platelet-derived growth factor (PDGFR/PDGF) system is a prognostic and treatment response biomarker with multifarious therapeutic targets in cancers. *Tumour Biol.* 37 10053–10066. 10.1007/s13277-016-5069-z 27193823

[B8] Asli N. R. Xaymardan M. Cornwell J. Forte E. Waardenberg A. J. Janbandhu V. (2017). PDGFRα signaling in cardiac fibroblasts modulates quiescence, metabolism and self-renewal, and promotes anatomical and functional repair. *bioRxiv* [Preprint] 2017/227979,

[B9] Asli N. S. Xaymardan M. Patrick M. Farbehi N. Cornwell J. Forte E. (2019). PDGFRα signaling in cardiac fibroblasts modulates quiescence, metabolism and self-renewal, and promotes anatomical and functional repair. *bioRxiv* [Preprint] 10.1101/225979

[B10] Astudillo P. (2020). Extracellular matrix stiffness and Wnt/beta-catenin signaling in physiology and disease. *Biochem. Soc. Trans.* 48 1187–1198. 10.1042/bst20200026 32412078

[B11] Awada H. K. Johnson N. R. Wang Y. (2015). Sequential delivery of angiogenic growth factors improves revascularization and heart function after myocardial infarction. *J. Control. Release* 207 7–17. 10.1016/j.jconrel.2015.03.034 25836592 PMC4430430

[B12] Battegay E. J. Rupp J. Iruela-Arispe L. Sage E. H. Pech M. (1994). PDGF-BB modulates endothelial proliferation and angiogenesis in vitro via PDGF beta-receptors. *J. Cell Biol.* 125 917–928. 10.1083/jcb.125.4.917 7514607 PMC2120083

[B13] Bax J. J. Schinkel A. F. Boersma E. Rizzello V. Elhendy A. Maat A. (2003). Early versus delayed revascularization in patients with ischemic cardiomyopathy and substantial viability: impact on outcome. *Circulation* 108(Suppl. 1) II39–II42. 12970206 10.1161/01.cir.0000089041.69175.9d

[B14] Bax N. A. Bleyl S. B. Gallini R. Wisse L. J. Hunter J. Van Oorschot A. A. (2010). Cardiac malformations in Pdgfralpha mutant embryos are associated with increased expression of WT1 and Nkx2.5 in the second heart field. *Dev. Dyn.* 239 2307–2317. 10.1002/dvdy.22363 20658695 PMC2911638

[B15] Bergmann O. Bhardwaj R. D. Bernard S. Zdunek S. Barnabe-Heider F. Walsh S. (2009). Evidence for cardiomyocyte renewal in humans. *Science* 324 98–102. 10.1126/science.1164680 19342590 PMC2991140

[B16] Bergsten E. Uutela M. Li X. Pietras K. Ostman A. Heldin C. H. (2001). PDGF-D is a specific, protease-activated ligand for the PDGF beta-receptor. *Nat. Cell Biol.* 3 512–516. 10.1038/35074588 11331881

[B17] Betsholtz C. (1995). Role of platelet-derived growth factors in mouse development. *Int. J. Dev. Biol.* 39 817–825. 8645566

[B18] Bjarnegard M. Enge M. Norlin J. Gustafsdottir S. Fredriksson S. Abramsson A. (2004). Endothelium-specific ablation of PDGFB leads to pericyte loss and glomerular, cardiac and placental abnormalities. *Development* 131 1847–1857. 10.1242/dev.01080 15084468

[B19] Bloomekatz J. Singh R. Prall O. W. Dunn A. C. Vaughan M. Loo C. S. (2017). Platelet-derived growth factor (PDGF) signaling directs cardiomyocyte movement toward the midline during heart tube assembly. *Elife* 6:e21172. 28098558 10.7554/eLife.21172 PMC5298878

[B20] Bottrell A. Meng Y. H. Najy A. J. Hurst N. Jr. Kim S. Kim C. J. (2019). An oncogenic activity of PDGF-C and its splice variant in human breast cancer. *Growth Factors* 37 131–145. 10.1080/08977194.2019.1662415 31542979 PMC6872946

[B21] Brown D. M. Hong S. P. Farrell C. L. Pierce G. F. Khouri R. K. (1995). Platelet-derived growth factor BB induces functional vascular anastomoses in vivo. *Proc. Natl. Acad. Sci. U.S.A.* 92 5920–5924. 10.1073/pnas.92.13.5920 7597054 PMC41613

[B22] Bruggemann T. Andresen D. Schroder R. (1989). [ST-segment analysis in long-term ECG: amplitude and phase response of various systems in comparison with standard ECG and their effect on true original reproduction of ST segment depression]. *Z. Kardiol.* 78 14–22. 2922953

[B23] Cao R. Brakenhielm E. Li X. Pietras K. Widenfalk J. Ostman A. (2002). Angiogenesis stimulated by PDGF-CC, a novel member in the PDGF family, involves activation of PDGFR-alphaalpha and -alphabeta receptors. *FASEB J.* 16 1575–1583. 10.1096/fj.02-0319com 12374780

[B24] Castellani C. Padalino M. China P. Fedrigo M. Frescura C. Milanesi O. (2010). Bone-marrow-derived CXCR4-positive tissue-committed stem cell recruitment in human right ventricular remodeling. *Hum. Pathol.* 41 1566–1576. 10.1016/j.humpath.2009.12.017 20621330

[B25] Charbonneau M. Lavoie R. R. Lauzier A. Harper K. McDonald P. P. Dubois C. M. (2016). Platelet-derived growth factor receptor activation promotes the prodestructive invadosome-forming phenotype of synoviocytes from patients with rheumatoid arthritis. *J. Immunol.* 196 3264–3275. 10.4049/jimmunol.1500502 26976956

[B26] Chen P. H. Chen X. He X. (2013). Platelet-derived growth factors and their receptors: structural and functional perspectives. *Biochim. Biophys. Acta* 1834 2176–2186. 10.1016/j.bbapap.2012.10.015 23137658 PMC3612563

[B27] Chiariello M. Marinissen M. J. Gutkind J. S. (2001). Regulation of c-myc expression by PDGF through Rho GTPases. *Nat. Cell Biol.* 3 580–586. 10.1038/35078555 11389443

[B28] Chintalgattu V. Ai D. Langley R. R. Zhang J. Bankson J. A. Shih T. L. (2010). Cardiomyocyte PDGFR-beta signaling is an essential component of the mouse cardiac response to load-induced stress. *J. Clin. Invest.* 120 472–484. 10.1172/jci39434 20071776 PMC2810076

[B29] Chong J. J. Chandrakanthan V. Xaymardan M. Asli N. S. Li J. Ahmed I. (2011). Adult cardiac-resident MSC-like stem cells with a proepicardial origin. *Cell Stem Cell* 9 527–540. 10.1016/j.stem.2011.10.002 22136928 PMC3652240

[B30] Chong J. J. Reinecke H. Iwata M. Torok-Storb B. Stempien-Otero A. Murry C. E. (2013). Progenitor cells identified by PDGFR-alpha expression in the developing and diseased human heart. *Stem Cells Dev.* 22 1932–1943. 10.1089/scd.2012.0542 23391309 PMC3685392

[B31] Davidson S. M. Ferdinandy P. Andreadou I. Botker H. E. Heusch G. Ibanez B. (2019). Multitarget strategies to reduce myocardial ischemia/reperfusion injury: JACC review topic of the week. *J. Am. Coll. Cardiol.* 73 89–99. 10.1016/j.jacc.2018.09.086 30621955

[B32] Davis D. R. (2019). Cardiac stem cells in the post-Anversa era. *Eur. Heart J.* 40 1039–1041. 10.1093/eurheartj/ehz098 30933292

[B33] De Marchis F. Ribatti D. Giampietri C. Lentini A. Faraone D. Scoccianti M. (2002). Platelet-derived growth factor inhibits basic fibroblast growth factor angiogenic properties in vitro and in vivo through its alpha receptor. *Blood* 99 2045–2053. 10.1182/blood.v99.6.2045 11877278

[B34] Ding G. Tanaka Y. Hayashi M. Nishikawa S. Kataoka H. (2013). PDGF receptor alpha+ mesoderm contributes to endothelial and hematopoietic cells in mice. *Dev. Dyn.* 242 254–268. 10.1002/dvdy.23923 23335233 PMC3597973

[B35] Ding H. Wu X. Kim I. Tam P. P. Koh G. Y. Nagy A. (2000). The mouse Pdgfc gene: dynamic expression in embryonic tissues during organogenesis. *Mech. Dev.* 96 209–213. 10.1016/s0925-4773(00)00425-1 10960785

[B36] Ehrlich H. P. Freedman B. M. (2002). Topical platelet-derived growth factor in patients enhances wound closure in the absence of wound contraction. *Cytokines Cell Mol. Ther.* 7 85–90. 10.1080/13684730310001643 12850807

[B37] Farahani R. M. Xaymardan M. (2015). Platelet-derived growth factor receptor alpha as a marker of mesenchymal stem cells in development and stem cell biology. *Stem Cells Int.* 2015:362753. 26257789 10.1155/2015/362753 PMC4519552

[B38] Farbehi N. Patrick R. Dorison A. Xaymardan M. Janbandhu V. Wystub-Lis K. (2019). Single-cell expression profiling reveals dynamic flux of cardiac stromal, vascular and immune cells in health and injury. *Elife* 8:e43882. 30912746 10.7554/eLife.43882 PMC6459677

[B39] Fazel S. Cimini M. Chen L. Li S. Angoulvant D. Fedak P. (2006). Cardioprotective c-kit+ cells are from the bone marrow and regulate the myocardial balance of angiogenic cytokines. *J. Clin. Invest.* 116 1865–1877. 10.1172/jci27019 16823487 PMC1483161

[B40] Fischer A. N. Fuchs E. Mikula M. Huber H. Beug H. Mikulits W. (2007). PDGF essentially links TGF-beta signaling to nuclear beta-catenin accumulation in hepatocellular carcinoma progression. *Oncogene* 26 3395–3405. 10.1038/sj.onc.1210121 17130832

[B41] Fredriksson L. Li H. Eriksson U. (2004). The PDGF family: four gene products form five dimeric isoforms. *Cytokine Growth Factor Rev.* 15 197–204. 10.1016/j.cytogfr.2004.03.007 15207811

[B42] Gallini R. Huusko J. Yla-Herttuala S. Betsholtz C. Andrae J. (2016a). Isoform-specific modulation of inflammation induced by adenoviral mediated delivery of platelet-derived growth factors in the adult mouse heart. *PLoS One* 11:e0160930. 10.1371/journal.pone.0160930 27513343 PMC4981378

[B43] Gallini R. Lindblom P. Bondjers C. Betsholtz C. Andrae J. (2016b). PDGF-A and PDGF-B induces cardiac fibrosis in transgenic mice. *Exp. Cell Res.* 349 282–290. 10.1016/j.yexcr.2016.10.022 27816607

[B44] Giugliano G. R. Giugliano R. P. Gibson C. M. Kuntz R. E. (2003). Meta-analysis of corticosteroid treatment in acute myocardial infarction. *Am. J. Cardiol.* 91 1055–1059. 10.1016/s0002-9149(03)00148-6 12714146

[B45] Gladh H. Folestad E. B. Muhl L. Ehnman M. Tannenberg P. Lawrence A. L. (2016). Mice lacking platelet-derived growth factor D display a mild vascular phenotype. *PLoS One* 11:e0152276. 10.1371/journal.pone.0152276 27032083 PMC4816573

[B46] Gowda S. Weinstein D. A. Blalock T. D. Gandhi K. Mast B. A. Chin G. (2015). Topical application of recombinant platelet-derived growth factor increases the rate of healing and the level of proteins that regulate this response. *Int. Wound J.* 12 564–571. 10.1111/iwj.12165 24118782 PMC7950648

[B47] Graham S. Leonidou A. Lester M. Heliotis M. Mantalaris A. Tsiridis E. (2009). Investigating the role of PDGF as a potential drug therapy in bone formation and fracture healing. *Expert Opin. Investig. Drugs* 18 1633–1654. 10.1517/13543780903241607 19747084

[B48] Harrington E. A. Bennett M. R. Fanidi A. Evan G. I. (1994). c-Myc-induced apoptosis in fibroblasts is inhibited by specific cytokines. *EMBO J.* 13 3286–3295. 10.1002/j.1460-2075.1994.tb06630.x 8045259 PMC395225

[B49] Hausenloy D. J. Yellon D. M. (2013). Myocardial ischemia-reperfusion injury: a neglected therapeutic target. *J. Clin. Invest.* 123 92–100. 10.1172/jci62874 23281415 PMC3533275

[B50] He C. Medley S. C. Hu T. Hinsdale M. E. Lupu F. Virmani R. (2015). PDGFRbeta signalling regulates local inflammation and synergizes with hypercholesterolaemia to promote atherosclerosis. *Nat. Commun.* 6:7770. 26183159 10.1038/ncomms8770 PMC4507293

[B51] Hellstrom M. Kalen M. Lindahl P. Abramsson A. Betsholtz C. (1999). Role of PDGF-B and PDGFR-beta in recruitment of vascular smooth muscle cells and pericytes during embryonic blood vessel formation in the mouse. *Development* 126 3047–3055. 10.1242/dev.126.14.3047 10375497

[B52] Hoch R. V. Soriano P. (2003). Roles of PDGF in animal development. *Development* 130 4769–4784. 10.1242/dev.00721 12952899

[B53] Horikawa S. Ishii Y. Hamashima T. Yamamoto S. Mori H. Fujimori T. (2015). PDGFRalpha plays a crucial role in connective tissue remodeling. *Sci. Rep.* 5:17948. 26639755 10.1038/srep17948 PMC4671150

[B54] Houlihan D. D. Mabuchi Y. Morikawa S. Niibe K. Araki D. Suzuki S. (2012). Isolation of mouse mesenchymal stem cells on the basis of expression of Sca-1 and PDGFR-α. *Nat. Protoc.* 7 2103–2111. 10.1038/nprot.2012.125 23154782

[B55] Hsieh P. C. Davis M. E. Gannon J. MacGillivray C. Lee R. T. (2006a). Controlled delivery of PDGF-BB for myocardial protection using injectable self-assembling peptide nanofibers. *J. Clin. Invest.* 116 237–248. 10.1172/jci25878 16357943 PMC1312017

[B56] Hsieh P. C. MacGillivray C. Gannon J. Cruz F. U. Lee R. T. (2006b). Local controlled intramyocardial delivery of platelet-derived growth factor improves postinfarction ventricular function without pulmonary toxicity. *Circulation* 114 637–644. 10.1161/circulationaha.106.639831 16894033

[B57] Ivey M. J. Tallquist M. D. (2016). Defining the cardiac fibroblast. *Circ. J.* 80 2269–2276. 10.1253/circj.cj-16-1003 27746422 PMC5588900

[B58] Kazlauskas A. (2017). PDGFs and their receptors. *Gene* 614 1–7. 10.1016/j.gene.2017.03.003 28267575 PMC6728141

[B59] Khoshkam V. Chan H. L. Lin G. H. Mailoa J. Giannobile W. V. Wang H. L. (2015). Outcomes of regenerative treatment with rhPDGF-BB and rhFGF-2 for periodontal intra-bony defects: a systematic review and meta-analysis. *J. Clin. Periodontol.* 42 272–280. 10.1111/jcpe.12354 25605424

[B60] Klinkhammer B. M. Floege J. Boor P. (2018). PDGF in organ fibrosis. *Mol. Aspects Med.* 62 44–62. 10.1016/j.mam.2017.11.008 29155002

[B61] Kong P. Christia P. Frangogiannis N. G. (2014). The pathogenesis of cardiac fibrosis. *Cell Mol. Life Sci.* 71 549–574. 23649149 10.1007/s00018-013-1349-6 PMC3769482

[B62] Kramann R. Machado F. Wu H. Kusaba T. Hoeft K. Schneider R. K. (2018). Parabiosis and single-cell RNA sequencing reveal a limited contribution of monocytes to myofibroblasts in kidney fibrosis. *JCI Insight* 3:e99561. 10.1172/jci.insight.99561 PMC6012505 29720573

[B63] Kramann R. Schneider R. K. DiRocco D. P. Machado F. Fleig S. Bondzie P. A. (2015). Perivascular Gli1+ progenitors are key contributors to injury-induced organ fibrosis. *Cell Stem Cell* 16 51–66. 10.1016/j.stem.2014.11.004 25465115 PMC4289444

[B64] Lee C. Li X. (2018). Platelet-derived growth factor-C and -D in the cardiovascular system and diseases. *Mol. Aspects Med.* 62 12–21. 10.1016/j.mam.2017.09.005 28965749

[B65] Lee C. Zhang F. Tang Z. Liu Y. Li X. (2013). PDGF-C: a new performer in the neurovascular interplay. *Trends Mol. Med.* 19 474–486. 10.1016/j.molmed.2013.04.006 23714575

[B66] Lee J. Termglinchan V. Diecke S. Itzhaki I. Lam C. K. Garg P. (2019). Activation of PDGF pathway links LMNA mutation to dilated cardiomyopathy. *Nature* 572 335–340. 10.1038/s41586-019-1406-x 31316208 PMC6779479

[B67] Lepisto J. Peltonen J. Vaha-Kreula M. Niinikoski J. Laato M. (1995). Platelet-derived growth factor isoforms PDGF-AA, -AB and -BB exert specific effects on collagen gene expression and mitotic activity of cultured human wound fibroblasts. *Biochem. Biophys. Res. Commun.* 209 393–399. 10.1006/bbrc.1995.1516 7733905

[B68] Leveen P. Pekny M. Gebre-Medhin S. Swolin B. Larsson E. Betsholtz C. (1994). Mice deficient for PDGF B show renal, cardiovascular, and hematological abnormalities. *Genes Dev.* 8 1875–1887. 10.1101/gad.8.16.1875 7958863

[B69] Li R. Bernau K. Sandbo N. Gu J. Preissl S. Sun X. (2018). Pdgfra marks a cellular lineage with distinct contributions to myofibroblasts in lung maturation and injury response. *Elife* 7:e36865. 30178747 10.7554/eLife.36865 PMC6122952

[B70] Li X. Ponten A. Aase K. Karlsson L. Abramsson A. Uutela M. (2000). PDGF-C is a new protease-activated ligand for the PDGF alpha-receptor. *Nat. Cell Biol.* 2 302–309. 10.1038/35010579 10806482

[B71] Libby P. Theroux P. (2005). Pathophysiology of coronary artery disease. *Circulation* 111 3481–3488. 15983262 10.1161/CIRCULATIONAHA.105.537878

[B72] Lindahl P. Johansson B. R. Leveen P. Betsholtz C. (1997). Pericyte loss and microaneurysm formation in PDGF-B-deficient mice. *Science* 277 242–245. 10.1126/science.277.5323.242 9211853

[B73] Liu C. Li J. Xiang X. Guo L. Tu K. Liu Q. (2014). PDGF receptor-alpha promotes TGF-beta signaling in hepatic stellate cells via transcriptional and posttranscriptional regulation of TGF-beta receptors. *Am. J. Physiol. Gastrointest. Liver Physiol.* 307 G749–G759. 25169976 10.1152/ajpgi.00138.2014 PMC4187064

[B74] Mahmoud A. I. Porrello E. R. Kimura W. Olson E. N. Sadek H. A. (2014). Surgical models for cardiac regeneration in neonatal mice. *Nat. Protoc.* 9 305–311. 10.1038/nprot.2014.021 24434799 PMC3977725

[B75] Mamer S. B. Chen S. Weddell J. C. Palasz A. Wittenkeller A. Kumar M. (2020). Author correction: discovery of high-affinity PDGF-VEGFR interactions: redefining RTK dynamics. *Sci. Rep.* 10:11001. 32601287 10.1038/s41598-020-63864-1 PMC7324393

[B76] Mellgren A. M. Smith C. L. Olsen G. S. Eskiocak B. Zhou B. Kazi M. N. (2008). Platelet-derived growth factor receptor beta signaling is required for efficient epicardial cell migration and development of two distinct coronary vascular smooth muscle cell populations. *Circ. Res.* 103 1393–1401. 10.1161/circresaha.108.176768 18948621 PMC2757035

[B77] Mohl W. Gangl C. Jusic A. Aschacher T. De Jonge M. Rattay F. (2015). PICSO: from myocardial salvage to tissue regeneration. *Cardiovasc. Revasc. Med.* 16 36–46. 10.1016/j.carrev.2014.12.004 25616738

[B78] Munoz-Chapuli R. Macias D. Gonzalez-Iriarte M. Carmona R. Atencia G. Perez-Pomares J. M. (2002). [The epicardium and epicardial-derived cells: multiple functions in cardiac development]. *Rev. Esp. Cardiol.* 55 1070–1082. 12383393 10.1016/s0300-8932(02)76758-4

[B79] Musa H. Kaur K. O’Connell R. Klos M. Guerrero-Serna G. Avula U. M. (2013). Inhibition of platelet-derived growth factor-AB signaling prevents electromechanical remodeling of adult atrial myocytes that contact myofibroblasts. *Heart Rhythm* 10 1044–1051. 10.1016/j.hrthm.2013.03.014 23499624 PMC3692578

[B80] Mustoe T. A. Cutler N. R. Allman R. M. Goode P. S. Deuel T. F. Prause J. A. (1994). A phase II study to evaluate recombinant platelet-derived growth factor-BB in the treatment of stage 3 and 4 pressure ulcers. *Arch. Surg.* 129 213–219. 10.1001/archsurg.1994.01420260109015 8304833

[B82] Okura T. Igase M. Kitami Y. Fukuoka T. Maguchi M. Kohara K. (1998). Platelet-derived growth factor induces apoptosis in vascular smooth muscle cells: roles of the Bcl-2 family. *Biochim. Biophys. Acta* 1403 245–253. 10.1016/s0167-4889(98)00065-2 9685664

[B83] Olsen R. S. Dimberg J. Geffers R. Wagsater D. (2019). Possible role and therapeutic target of PDGF-D signalling in colorectal cancer. *Cancer Invest.* 37 99–112. 10.1080/07357907.2019.1576191 30836770

[B84] Olson L. E. Soriano P. (2009). Increased PDGFRalpha activation disrupts connective tissue development and drives systemic fibrosis. *Dev. Cell* 16 303–313. 10.1016/j.devcel.2008.12.003 19217431 PMC2664622

[B85] Orr-Urtreger A. Bedford M. T. Do M. S. Eisenbach L. Lonai P. (1992). Developmental expression of the alpha receptor for platelet-derived growth factor, which is deleted in the embryonic lethal Patch mutation. *Development* 115 289–303. 10.1242/dev.115.1.289 1322271

[B86] Ostman A. (2004). PDGF receptors-mediators of autocrine tumor growth and regulators of tumor vasculature and stroma. *Cytokine Growth Factor Rev.* 15 275–286. 10.1016/j.cytogfr.2004.03.002 15207817

[B87] Palmieri S. L. Payne J. Stiles C. D. Biggers J. D. Mercola M. (1992). Expression of mouse PDGF-A and PDGF alpha-receptor genes during pre- and post-implantation development: evidence for a developmental shift from an autocrine to a paracrine mode of action. *Mech. Dev.* 39 181–191. 10.1016/0925-4773(92)90045-l 1292572

[B88] Papadopoulos N. Lennartsson J. (2018). The PDGF/PDGFR pathway as a drug target. *Mol. Aspects Med.* 62 75–88. 10.1016/j.mam.2017.11.007 29137923

[B89] Pierce G. F. Tarpley J. E. Tseng J. Bready J. Chang D. Kenney W. C. (1995). Detection of platelet-derived growth factor (PDGF)-AA in actively healing human wounds treated with recombinant PDGF-BB and absence of PDGF in chronic nonhealing wounds. *J. Clin. Invest.* 96 1336–1350. 10.1172/jci118169 7657809 PMC185756

[B90] Ponten A. Folestad E. B. Pietras K. Eriksson U. (2005). Platelet-derived growth factor D induces cardiac fibrosis and proliferation of vascular smooth muscle cells in heart-specific transgenic mice. *Circ. Res.* 97 1036–1045. 10.1161/01.res.0000190590.31545.d4 16224065

[B91] Ponten A. Li X. Thoren P. Aase K. Sjoblom T. Ostman A. (2003). Transgenic overexpression of platelet-derived growth factor-C in the mouse heart induces cardiac fibrosis, hypertrophy, and dilated cardiomyopathy. *Am. J. Pathol.* 163 673–682. 10.1016/s0002-9440(10)63694-2 12875986 PMC1868211

[B92] Porrello E. R. Mahmoud A. I. Simpson E. Hill J. A. Richardson J. A. Olson E. N. (2011). Transient regenerative potential of the neonatal mouse heart. *Science* 331 1078–1080. 10.1126/science.1200708 21350179 PMC3099478

[B93] Prall O. W. Menon M. K. Solloway M. J. Watanabe Y. Zaffran S. Bajolle F. (2007). An Nkx2-5/Bmp2/Smad1 negative feedback loop controls heart progenitor specification and proliferation. *Cell* 128 947–959. 10.1016/j.cell.2007.01.042 17350578 PMC2092439

[B94] Price R. L. Haley S. T. Bullard T. A. Goldsmith E. C. Simpson D. G. Thielen T. E. (2003). Effects of platelet-derived growth factor-AA and -BB on embryonic cardiac development. *Anat. Rec. A Discov. Mol. Cell. Evol. Biol.* 272 424–433. 10.1002/ar.a.10054 12704700

[B95] Qian C. Wong C. W. Y. Wu Z. He Q. Xia H. Tam P. K. H. (2017). Stage specific requirement of platelet-derived growth factor receptor-alpha in embryonic development. *PLoS One* 12:e0184473. 10.1371/journal.pone.0184473 28934221 PMC5608218

[B96] Qian C. Wu Z. Ng R. C. Garcia-Barcelo M. M. Yuan Z. W. Wong K. K. Y. (2019). Conditional deletion of platelet derived growth factor receptor alpha (Pdgfra) in urorectal mesenchyme causes mesenchyme apoptosis and urorectal developmental anomalies in mice. *Cell Death Differ.* 26 1396–1410. 10.1038/s41418-018-0216-2 30323271 PMC6748092

[B97] Quijada P. Trembley M. A. Small E. M. (2020). The Role of the epicardium during heart development and repair. *Circ. Res.* 126 377–394. 10.1161/circresaha.119.315857 31999538 PMC7000171

[B98] Ramos I. T. Henningsson M. Nezafat M. Lavin B. Lorrio S. Gebhardt P. (2018). Simultaneous assessment of cardiac inflammation and extracellular matrix remodeling after myocardial infarction. *Circ. Cardiovasc. Imaging* 11:e007453. 30524648 10.1161/CIRCIMAGING.117.007453 PMC6277008

[B99] Ranganath S. H. Levy O. Inamdar M. S. Karp J. M. (2012). Harnessing the mesenchymal stem cell secretome for the treatment of cardiovascular disease. *Cell Stem Cell* 10 244–258. 10.1016/j.stem.2012.02.005 22385653 PMC3294273

[B100] Roehlen N. Crouchet E. Baumert T. F. (2020). Liver fibrosis: mechanistic concepts and therapeutic perspectives. *Cells* 9:875. 10.3390/cells9040875 32260126 PMC7226751

[B101] Romashkova J. A. Makarov S. S. (1999). NF-kappaB is a target of AKT in anti-apoptotic PDGF signalling. *Nature* 401 86–90. 10.1038/43474 10485711

[B102] Santini M. P. Malide D. Hoffman G. Pandey G. D’Escamard V. Nomura-Kitabayashi A. (2020). Tissue-resident PDGFRalpha(+) progenitor cells contribute to fibrosis versus healing in a context- and spatiotemporally dependent manner. *Cell Rep.* 30 555–570.e7. 31940496 10.1016/j.celrep.2019.12.045 PMC7030884

[B103] Sebastiao M. J. Pereira R. Serra M. Gomes-Alves P. Alves P. M. (2018). Unveiling human cardiac fibroblast membrane proteome. *Proteomics* 18:e1700446. 29696784 10.1002/pmic.201700446

[B104] Smith C. L. Baek S. T. Sung C. Y. Tallquist M. D. (2011). Epicardial-derived cell epithelial-to-mesenchymal transition and fate specification require PDGF receptor signaling. *Circ. Res.* 108 e15–e26. 21512159 10.1161/CIRCRESAHA.110.235531 PMC3134964

[B105] Soriano P. (1997). The PDGF alpha receptor is required for neural crest cell development and for normal patterning of the somites. *Development* 124 2691–2700. 10.1242/dev.124.14.2691 9226440

[B106] Souders C. A. Bowers S. L. Baudino T. A. (2009). Cardiac fibroblast: the renaissance cell. *Circ. Res.* 105 1164–1176. 10.1161/circresaha.109.209809 19959782 PMC3345531

[B107] Su W. Liu G. Liu X. Zhou Y. Sun Q. Zhen G. (2020). Angiogenesis stimulated by elevated PDGF-BB in subchondral bone contributes to osteoarthritis development. *JCI Insight* 5:e135446. 10.1172/jci.insight.135446 PMC7205438 32208385

[B108] Sullivan K. E. Quinn K. P. Tang K. M. Georgakoudi I. Black L. D. III (2014). Extracellular matrix remodeling following myocardial infarction influences the therapeutic potential of mesenchymal stem cells. *Stem Cell Res. Ther.* 5:14. 24460869 10.1186/scrt403 PMC4055039

[B109] Taimeh Z. Loughran J. Birks E. J. Bolli R. (2013). Vascular endothelial growth factor in heart failure. *Nat. Rev. Cardiol.* 10 519–530. 23856679 10.1038/nrcardio.2013.94

[B110] Tallquist M. D. Soriano P. (2003). Cell autonomous requirement for PDGFRalpha in populations of cranial and cardiac neural crest cells. *Development* 130 507–518. 10.1242/dev.00241 12490557

[B111] Thavapalachandran S. Grieve S. M. Hume R. D. Le T. Y. L. Raguram K. Hudson J. E. (2020). Platelet-derived growth factor-AB improves scar mechanics and vascularity after myocardial infarction. *Sci. Transl. Med.* 12:eaay2140. 10.1126/scitranslmed.aay2140 31894101

[B112] Travis T. E. Mauskar N. A. Mino M. J. Prindeze N. Moffatt L. T. Fidler P. E. (2014). Commercially available topical platelet-derived growth factor as a novel agent to accelerate burn-related wound healing. *J. Burn Care Res.* 35 e321–e329. 24476989 10.1097/BCR.0000000000000013

[B113] Uosaki H. Andersen P. Shenje L. T. Fernandez L. Christiansen S. L. Kwon C. (2012). Direct contact with endoderm-like cells efficiently induces cardiac progenitors from mouse and human pluripotent stem cells. *PLoS One* 7:e46413. 10.1371/journal.pone.0046413 23056302 PMC3462754

[B114] Valius M. Kazlauskas A. (1993). Phospholipase C-gamma 1 and phosphatidylinositol 3 kinase are the downstream mediators of the PDGF receptor’s mitogenic signal. *Cell* 73 321–334. 10.1016/0092-8674(93)90232-f 7682895

[B115] Van den Akker N. M. Winkel L. C. Nisancioglu M. H. Maas S. Wisse L. J. Armulik A. (2008). PDGF-B signaling is important for murine cardiac development: its role in developing atrioventricular valves, coronaries, and cardiac innervation. *Dev. Dyn.* 237 494–503. 10.1002/dvdy.21436 18213589

[B116] Vantler M. Karikkineth B. C. Naito H. Tiburcy M. Didie M. Nose M. (2010). PDGF-BB protects cardiomyocytes from apoptosis and improves contractile function of engineered heart tissue. *J. Mol. Cell. Cardiol.* 48 1316–1323. 10.1016/j.yjmcc.2010.03.008 20307544

[B117] Wang Y. Appiah-Kubi K. Wu M. Yao X. Qian H. Wu Y. (2016). The platelet-derived growth factors (PDGFs) and their receptors (PDGFRs) are major players in oncogenesis, drug resistance, and attractive oncologic targets in cancer. *Growth Factors* 34 64–71. 10.1080/08977194.2016.1180293 27170215

[B118] Weisman H. F. Healy B. (1987). Myocardial infarct expansion, infarct extension, and reinfarction: pathophysiologic concepts. *Prog. Cardiovasc. Dis.* 30 73–110. 10.1016/0033-0620(87)90004-1 2888158

[B119] White S. J. Chong J. J. H. (2020). Growth factor therapy for cardiac repair: an overview of recent advances and future directions. *Biophys. Rev.* 12 805–815. 10.1007/s12551-020-00734-0 32691300 PMC7429584

[B120] Wills A. A. Holdway J. E. Major R. J. Poss K. D. (2008). Regulated addition of new myocardial and epicardial cells fosters homeostatic cardiac growth and maintenance in adult zebrafish. *Development* 135 183–192. 10.1242/dev.010363 18045840

[B121] Wollin L. Maillet I. Quesniaux V. Holweg A. Ryffel B. (2014). Antifibrotic and anti-inflammatory activity of the tyrosine kinase inhibitor nintedanib in experimental models of lung fibrosis. *J. Pharmacol. Exp. Ther.* 349 209–220. 10.1124/jpet.113.208223 24556663

[B122] Xaymardan M. Zheng J. Duignan I. Chin A. Holm J. M. Ballard V. L. (2004). Senescent impairment in synergistic cytokine pathways that provide rapid cardioprotection in the rat heart. *J. Exp. Med.* 199 797–804. 10.1084/jem.20031639 15007092 PMC2212728

[B123] Xu J. Clark R. A. (1996). Extracellular matrix alters PDGF regulation of fibroblast integrins. *J. Cell Biol.* 132 239–249. 10.1083/jcb.132.1.239 8567727 PMC2120701

[B124] Ying H. Z. Chen Q. Zhang W. Y. Zhang H. H. Ma Y. Zhang S. Z. (2017). PDGF signaling pathway in hepatic fibrosis pathogenesis and therapeutics (Review). *Mol. Med. Rep.* 16 7879–7889. 10.3892/mmr.2017.7641 28983598 PMC5779870

[B125] Yla-Herttuala S. Bridges C. Katz M. G. Korpisalo P. (2017). Angiogenic gene therapy in cardiovascular diseases: dream or vision? *Eur. Heart J.* 38 1365–1371. 28073865 10.1093/eurheartj/ehw547 PMC5837788

[B126] Yoon C. Song H. Yin T. Bausch-Fluck D. Frei A. P. Kattman S. (2018). FZD4 marks lateral plate mesoderm and signals with NORRIN to increase cardiomyocyte induction from pluripotent stem cell-derived cardiac progenitors. *Stem Cell Rep.* 10 87–100. 10.1016/j.stemcr.2017.11.008 29249665 PMC5768897

[B127] Yue Z. Chen J. Lian H. Pei J. Li Y. Chen X. (2019). PDGFR-beta signaling regulates cardiomyocyte proliferation and myocardial regeneration. *Cell Rep.* 28 966–978.e4. 31340157 10.1016/j.celrep.2019.06.065

[B128] Zhang J. Cao R. Zhang Y. Jia T. Cao Y. Wahlberg E. (2009). Differential roles of PDGFR-alpha and PDGFR-beta in angiogenesis and vessel stability. *FASEB J.* 23 153–163. 10.1096/fj.08-113860 18827023

[B129] Zhao T. Zhao W. Chen Y. Li V. S. Meng W. Sun Y. (2013). Platelet-derived growth factor-D promotes fibrogenesis of cardiac fibroblasts. *Am. J. Physiol. Heart Circ. Physiol.* 304 H1719–H1726. 23585135 10.1152/ajpheart.00130.2013 PMC3680773

[B130] Zhao W. Zhao T. Huang V. Chen Y. Ahokas R. A. Sun Y. (2011). Platelet-derived growth factor involvement in myocardial remodeling following infarction. *J. Mol. Cell. Cardiol.* 51 830–838. 10.1016/j.yjmcc.2011.06.023 21767547 PMC3628689

[B131] Zheng J. Shin J. H. Xaymardan M. Chin A. Duignan I. Hong M. K. (2004). Platelet-derived growth factor improves cardiac function in a rodent myocardial infarction model. *Coron. Artery Dis.* 15 59–64. 10.1097/00019501-200402000-00009 15201622

[B132] Zhou X. M. Liu Y. Payne G. Lutz R. J. Chittenden T. (2000). Growth factors inactivate the cell death promoter BAD by phosphorylation of its BH3 domain on Ser155. *J. Biol. Chem.* 275 25046–25051. 10.1074/jbc.m002526200 10837473

[B133] Zymek P. Bujak M. Chatila K. Cieslak A. Thakker G. Entman M. L. (2006). The role of platelet-derived growth factor signaling in healing myocardial infarcts. *J. Am. Coll. Cardiol.* 48 2315–2323. 10.1016/j.jacc.2006.07.060 17161265

